# Polymer Networks Produced by Marine Diatoms in the Northern Adriatic Sea

**DOI:** 10.3390/md9040666

**Published:** 2011-04-21

**Authors:** Vesna Svetličić, Vera Žutić, Tea Mišić Radić, Galja Pletikapić, Amela Hozić Zimmermann, Ranieri Urbani

**Affiliations:** 1 Division for Marine and Environmental Research, Ruđer Bošković Institute, Bijenička 54, Zagreb, Croatia; E-Mails: zutic@irb.hr (V. Ž.); tmisic@irb.hr (T.M.R.); gpletik@irb.hr (G.P.); ahozic@irb.hr (A.H.Z.); 2 Department of Life Sciences, University of Trieste, I-34127 Trieste, Italy; E-Mail: rurbani@units.it

**Keywords:** *Cylindrotheca closterium*, atomic force microscopy, Northern Adriatic macroaggregates, extracellular polysaccharides, marine gel network, self-assembly

## Abstract

Using high resolution molecular technique of atomic force microscopy, we address the extracellular polymer production of Adriatic diatom *Cylindrotheca closterium* analyzed at the single cell level and the supramolecular organization of gel phase isolated from the Northern Adriatic macroaggregates. Our results revealed that extracellular polysaccharides freshly produced by marine diatoms can self-assemble directly to form gel network characteristics of the macroscopic gel phase in the natural aquatorium. Based on the experiments performed with isolated polysaccharide fractions of *C. closterium* and of macroaggregates gel phase, we demonstrated that the polysaccharide self-assembly into gel network can proceed independent of any bacterial mediation or interaction with inorganic particles.

## 1. Introduction

**Figure 1.**
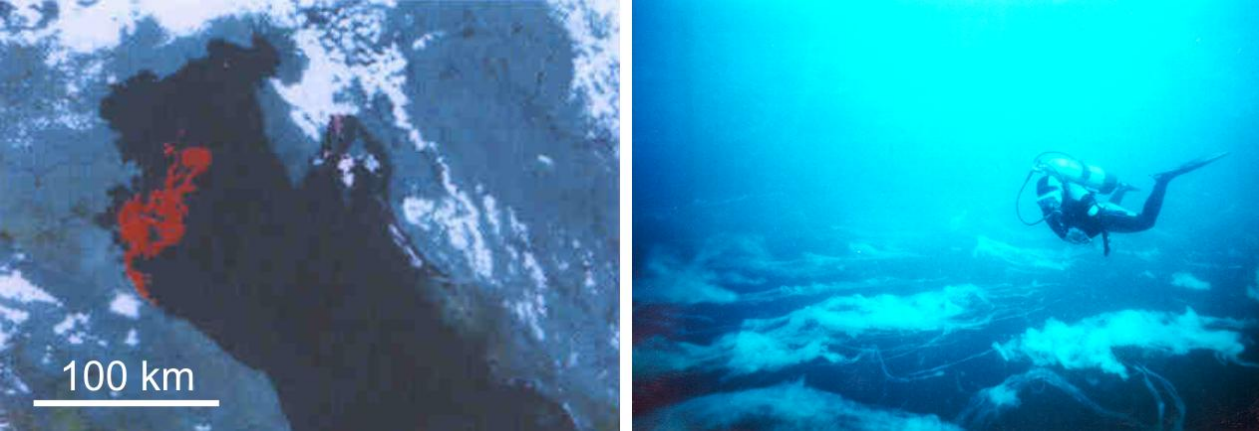
Northern Adriatic gel aggregates: (**a**) remote sensing by satellite showing gel phase in red color (reproduced from Zambianchi, E. *et al.* 1992 with kind permission from Elsevier B.V.) [3]; and (**b**) at 10 m depth captured by a scuba-diver in August 1997 (courtesy of Gerald Müller-Niklas).

The most intensive primary production and DOM transformation in the Mediterranean basin takes place in the northern part of the Adriatic Sea [1]. Eutrophication of the Northern Adriatic [2,3], due to the run-off from the Po and smaller rivers, causes hyper-production of phytoplankton during spring/summer at rates which far exceed the grazing potential of herbivores or rate of decomposition by bacteria [4]. Consequently, large standing stocks of phytoplankton build up and extracellular polymers, mainly polysaccharides, accumulate in the water column, especially in the euphotic layer above the picnocline. The enigmatic gel phase (mucilage) appears episodically in the northern Adriatic Sea (Figure 1). The phenomenon manifests itself in rapid production of enormous amounts of gelatinous matter in the water column and on the sea surface. The increased intensity and frequency of the phenomenon coincided with the phosphate ban in detergents in the eighties [5] and the diatom dominance in phytoplankton populations as a consequence. A relationship between phytoplankton biomass, dissolved carbohydrate concentrations and mucilage episodes is at present still not clarified [6–9]. Many studies have already been published that relate diatom extracellular polymer production with the phenomenon of macroscopic gel formation in the northern Adriatic Sea, in particular diatom species *Cylindrotheca closterium* [10–12], which is being regularly observed as a dominant species in the macroaggregates [11,13,14]. Chemical characterization of diatom extracellular polymeric substance (EPS) isolated from laboratory cultures [15–18] revealed that extracellular polymers are predominantly polysaccharides that contain substantial amounts of uronic acid and sulfate residues and may contain proteins in the form of proteoglycans or glycoproteins [19]. Sulfates present in EPS have the capacity to hold water molecules that play an important role in imparting gel-like consistency to the EPS [20].

The diatom produced polymers can self-assemble to form macroscopic gel phase [21,22]. The time that elapses from the diatom bloom to the gel formation is approximately 2–3 months, depending on many factors/conditions like temperature, stratification in the water column, salinity, current regime, and input of freshwater from the Po River. The question is whether bacterial modification of photosynthetically produced polymers is necessary prior to the gel formation [23] and if the mineral nanoparticles (colloids) are needed for gelation to proceed [24]. Three different scenarios could be foreseen: (i) photosynthetically produced polymers have the capacity to self-assemble into macroscopic gel phase without bacterial transformation or interaction with mineral particles; (ii) photosynthetically produced polymers undergo bacterial transformation prior to gel formation; (iii) photosynthetically produced polymers adsorb on mineral nanoparticles that act as nucleation centers for the gelation to proceed.

Those questions can be resolved using high resolution imaging technique of atomic force microscopy (AFM) to visualize polymer networks produced by marine diatom in the culture and networks of macroscopic gel phase formed during the “mucilage” episodes in the northern Adriatic basin. Here we address the extracellular polymer production of *C. closterium* isolated from the northern Adriatic Sea. Polysaccharide fraction isolated from cell culture and from macroscopic gel phase will be used to compare the capacities for network formation without the influence of bacterial action and without presence of mineral particles as nucleation sites.

## 2. Results and Discussion

Atomic force microscopy may provide new information on complex heterogeneous structures, offering high spatial resolution in three dimensions, down to subnanometer scale, while operating under ambient conditions [25–28]. Polysaccharide samples for AFM imaging [25,29–31] and polysaccharide gels [25,32–36] are usually spread on freshly cleaved mica surface. The imaging of hydrated samples is preferably conducted in air to inhibit the unfavorable motion of polysaccharides in liquid medium. Such AFM studies have been validated against data obtained directly under buffers, TEM studies and cryo-AFM. Balnois and Wilkinson [37] showed that when AFM is operated under ambient conditions, the thin water layer both sorbed to the biopolymers and presented on the mica surface maintaining molecular structure during AFM imaging.

### 2.1. Extracellular Polymers Released by Cylindrotheca Closterium

**Figure 2.**
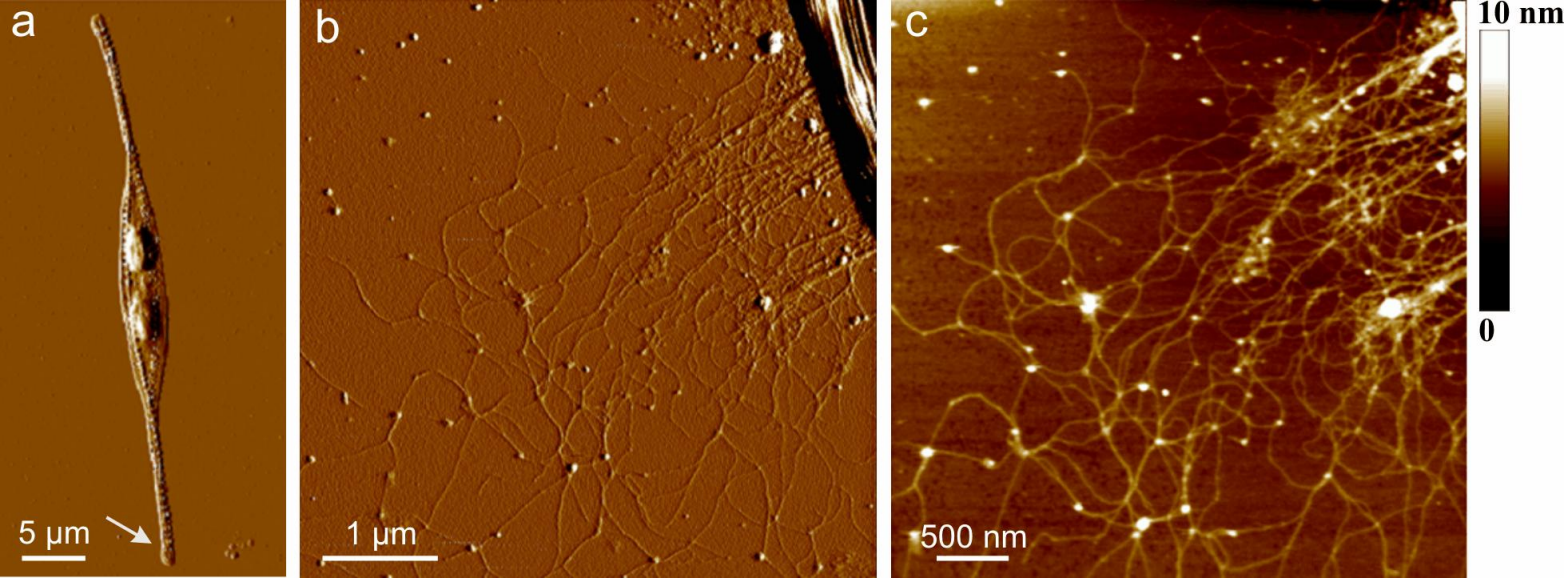
Extracellular polymers released by *C. closterium* obtained by Atomic force microscopy (AFM) imaging in contact mode after deposition on mica surface. (**a**) AFM image of the whole cell presented as deflection data. Arrow indicates the position of the polymer excretion site; (**b**) The released polymers still attached to the apex of the cell rostrum, deflection data, scan size 5 μm × 5 μm; (**c**) Released polymers presented as height data, scan size 4 μm × 4 μm and vertical scale shown as the color bar.

Figure 2 shows AFM image of extracellular polymers released by *C. closterium*. Figure 2a revealed the general features of a live *C. closterium* cell with the two chloroplasts and its drawn-out flexible rostra. Arrow indicates the position of polymer release shown in Figures b and c. Continuous scans were performed over the same region (20 times, slow scan: 1 Hz, 512 samples) and the structure shown was not altered. Parallel experiments with Alcian Blue staining performed in cell culture and light microscopy have shown that the polymers extending from the cell rostrum are mainly polysaccharides and existed before the cell deposition to the mica surface. The spatial arrangement of the polymers might be to a certain extent distorted from the three-dimensional structure in the aqueous phase due to the attachment and spreading on the mica surface. The bundles of polymer fibrils extended up to 10 μm from the cell surface. Their heights are 5–7 nm at the position close to the site of excretion. At a distance of 1 μm the dense network is observed with fibril heights of 2–3 nm. At even larger distances the network is less dense with the fibril heights in the range of 0.6 to 1.2 nm. The distribution of fibril heights is given in Table 1. The lower value of fibril height corresponds to the single monomolecular polysaccharide chains [30]. At this larger distance, the network appeared with incorporated spherical nanoparticles–globules. The globules are found to interconnect two or more fibrils. The globules may represent positively charged proteins whose function before the release is efficient intracellular packing of negatively charged polysaccharide fibrils, in line with molecular crowding in living cells [22].

**Figure 3.**
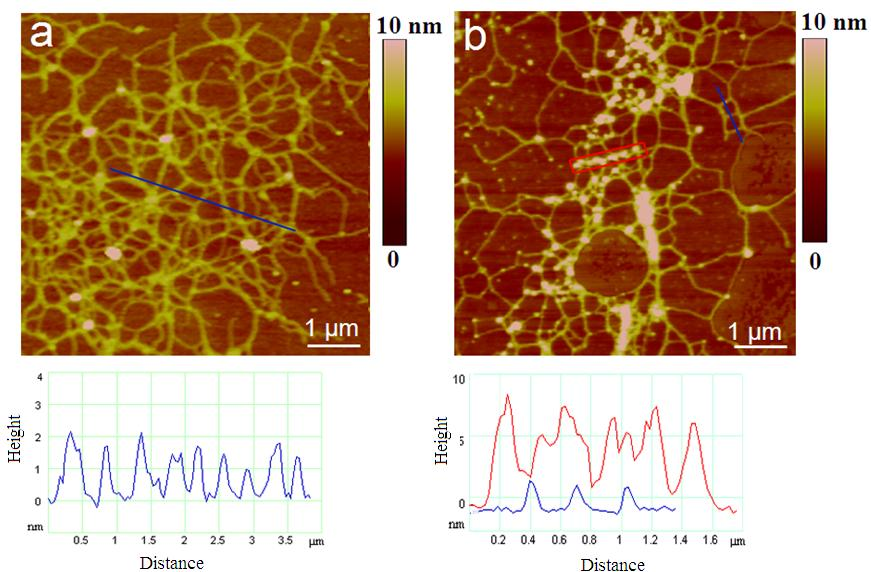
AFM images of polymer network from the bulk medium of *C. closterium* culture: (**a**) three-dimensional polymer network together with height profile of fibrils along the indicated line; (**b**) polymer network with attached particles and height profiles of fibrils and particles. Images are acquired in contact mode and presented as height data and the scan size is 7 μm × 7 μm.

Extracellular polymers released in culture medium, when adsorbed on mica surface, appear as single fibrils, but also as a collapsed three-dimensional network. The fibrils height range was 0.4–2.6 nm. Figure 3 shows two examples of polymer networks, one with high degree of fibril associations (Figure 3a) and the other with low degree of fibril associations and with a number of particles attached along fibrils (Figure 3b). The particles in Figure 3b are significantly larger (see corresponding vertical profiles) and are positioned mainly along the fibrils in contrast to the globules in Figure 2. The particles could be proteinic globules or organo-silica formed by interaction of silica-rich medium with polysaccharide network [38]. Interestingly, the network with a high degree of fibril associations has only a few incorporated particles.

### 2.2. Polymer Networks of Marine Gel

**Figure 4.**
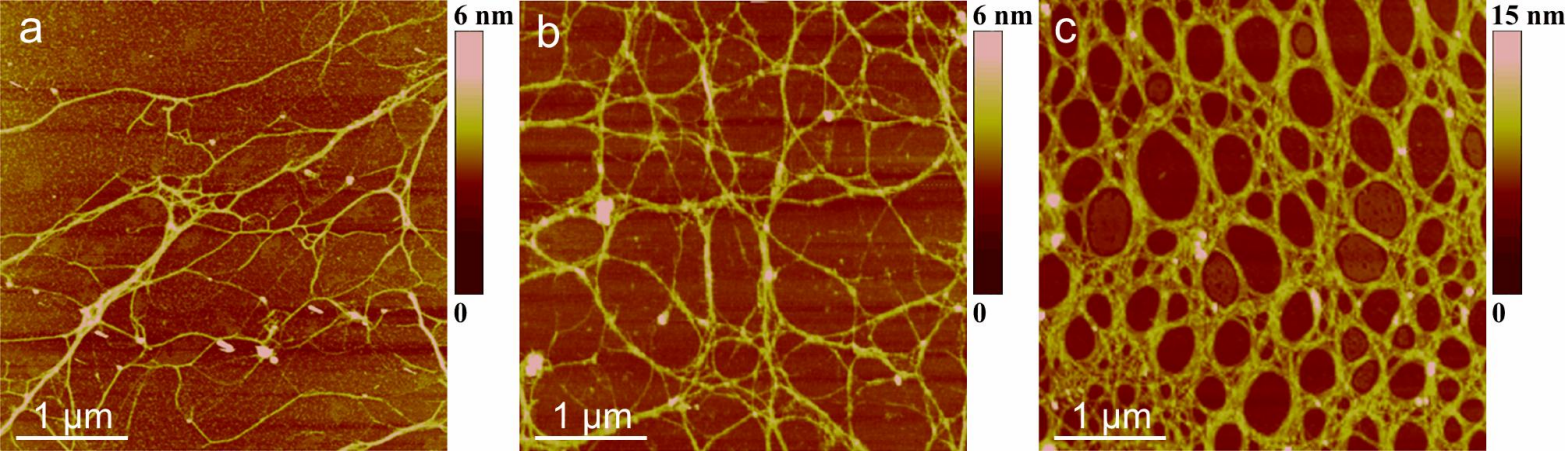
Evolution of polymer networks in the macroscopic gel phase from (**a**) to (**c**): early stage of gel phase formation (a) to condensed gel network of older macroaggregate (c). AFM images are acquired in contact mode and presented as height data, scan size 4 μm ×4 μm.

Figure 4 represents the evolution of polymer networks in the macroscopic gel phase. Samples were prepared from the macroaggregates with different residence time in the water column, from early stage of gel phase formation to the condensed (mature) gel network of an older macroaggregate.

The long polymer strands with small patches of initial fibril associations (Figure 4a) coexisted with the continuous gel network shown in Figure 4b. With the prolonged residence time (one month) the more condensed network is formed as presented in Figure 4c. The analysis of fibril heights for early and mature gel state is given in Table 1. The fibril heights for the early stage of gel network correspond to the fibril heights produced by Adriatic *C. closterium*.

**Figure 5.**
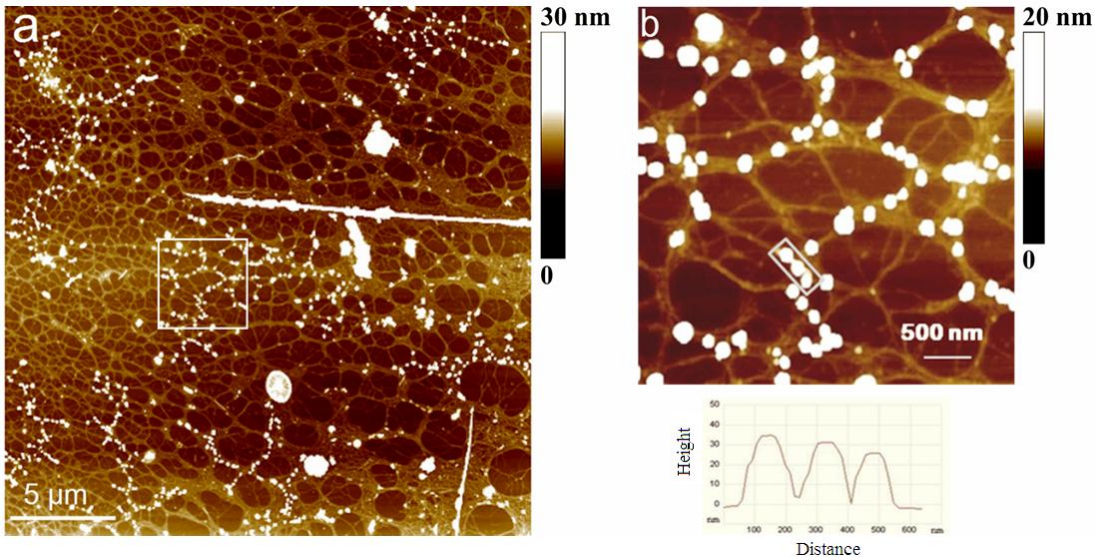
The pattern of nanoparticles distribution in gel network of macroaggregate sample: (**a**) scan size 25 μm × 25 μm and (**b**) indicated area imaged at higher resolution (scan size 4 μm × 4 μm) together with the height profile of particles. AFM images are acquired in contact mode and presented as height data.

When imaging large areas of gel network (e.g., scan size 25 μm × 25 μm) we occasionally encountered regions populated by numerous nanoparticles “sitting” on the fibrils (Figure 5a). The other objects were identified as body scales and diatom debris. Interestingly, nanoparticles were not found as free standing entities. The region of network bearing nanoparticles is illustrated in Figure 5b together with the height profile of indicated particles. The particles are not evenly distributed and do not appear as nucleation centers for network formation [39] and were most likely attached after the formation of gel phase. The particles are likely to represent biogenic silica originating from degraded diatom frustules. Numerous observations in different diatom species demonstrate that silica structures are formed of 2–200 nm diameter spherical particles [40–42].

### 2.3. Macromolecular Properties of the Isolated Polysaccharide Fraction of Marine Gel

Purified polysaccharides PS1 and PS2 obtained from two different mucilage events (2000 and 2001) were characterized using physicochemical approach in order to elucidate those macromolecular properties which strongly influence the chain propensity to form aggregates or gel-like structures in aqueous salt solution. These properties are the average chain dimension expressed by the radius of gyration (R_G_) and the weight-average molecular weight (M_w_), the second virial coefficient (A_2_) and the stiffness parameter (B) that are related to the propensity of the polymer system to give elongated and stiff chain.

From the linear dependence of intrinsic viscosity on the reciprocal ionic strength, measured by capillary viscometry on dilute polysaccharide solutions, the Smidsrød-Haug parameter B was calculated and chosen as characteristic of chain stiffness [43,44]. In general, the more flexible the chain, the higher the B value. The calculated B value 0.036 is relatively low, comparable to that of semi-rigid alginate chain. In general, going from flexible polysaccharides toward more rigid and extended chains, there is a general propensity of polymer chains to form chain associations or multiple helical structures.

Considering laser light scattering results, simultaneous linear least squares fits to both the angular and concentration dependence of scattering intensities were employed in the Zimm plot analysis [45]. By data fitting, PS1 and PS2 polysaccharides in solution showed a similar M_w_ value of about 230 kDa but different chain extensions as is revealed by the radius of gyration R_G_ (Table 2).

The results in Table 2 show, in addition, a constancy of R_G_ and M_W_ at different salt concentrations, while A_2_ becomes more negative as the screening effect of added salt on polymer charges increases. The polysaccharides analyzed here possess higher R_G_ values with respect to the flexible and coiled chains of pullulan and to the semi-rigid chain of the bacterial polysaccharide wellan [46] or alginate. The greater dimension of PS2 chains (R_G_ = 131–155 nm) with respect to the PS1 ones (R_G_ = 77–99 nm) reflects their higher charge density which amounts to 18% of uronic and sulfate groups with respect to the 10% of the latter [47]. These features confer a marked polyelectrolytic behavior and an expansion of the chain by charge repulsion [48], allowing a major solubility of polysaccharides in salt solutions and, at a given salt concentration, favoring chain aggregation and/or gel formation. Moreover the rather high decrease of A_2_ when exposed to higher ionic strengths (from 0.1 to 0.7 M NaCl) is the evidence of an extensive degree of aggregation in these conditions. A similar behavior has been also reported for polysaccharides extracted from benthic mucilagineous aggregates produced by *Acinetospora crinita* and *Chrysonephos lewisii* macroalga [49]. These results suggest a mechanism of aggregate formation which strongly depends, in addition to other environmental parameters, on the sea water salinity. It is noticeable that very often the first appearance of aggregates in water column is observed at the halocline strong salinity gradient where dissolved biopolymers reach a critical concentration and may aggregate by the increasing salt condition. In conclusion, our results reveal the mucilage polysaccharides in solution behave as rigid, very extended and aggregating polymers which tend to form fibrilar structures (typical diameter between 1 and 3 nm and length of 100–2000 nm) of physically cross-linked macromolecules.

### 2.4. Polysaccharide Fraction Isolated from Cell Culture

**Figure 6.**
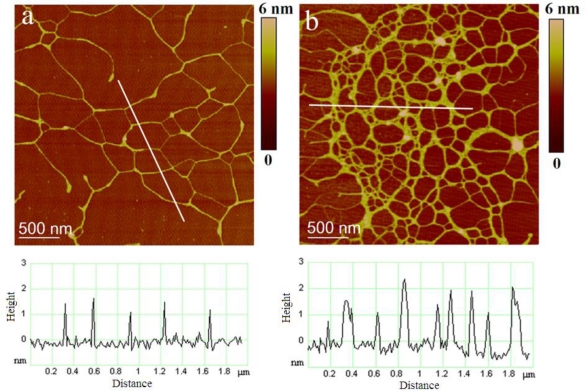
AFM images of polysaccharides isolated from the cell culture and dissolved in ultrapure water (concentration 10 mg/L) together with height profile of fibrils along indicated lines: (**a**) primary gel network and (**b**) network with high degree of fibril associations. Images are acquired in tapping mode and are presented as height data, scan size 3 μm × 3 μm.

The polysaccharide fraction isolated from the axenic *C. closterium* culture medium was used to test the capacity of photosynthetically produced polymers to self-assemble into gel phase without undergoing bacterial transformation. AFM imaging of samples prepared from the solution containing 10 mg/L revealed fibrilar networks varying in the degree of fibril associations (Figure 6). Figure 6a represents the primary gel network with fibril heights of 0.5–1.8 nm. Segment forming the network shown in Figure 6b are significantly wider with fibril heights in the range of 0.9 to 2.6 nm.

The fact that the isolated polysaccharide fraction has the capacity to self-assemble into a gel network in pure water is an important finding with implications on the mechanism of the macroscopic gel phase formation in the Northern Adriatic Sea. The AFM imaging of marine gel samples collected in summers 2003 and 2004 in the northern Adriatic Sea [21] provided insight into molecular organization of gel network and associations between polysaccharide fibrils in the network. The marine gel is characterized as a thermoreversible physical gel and the dominant mode of gelation as crosslinking of polysaccharide fibrils by hydrogen bonding which results in helical structures and their associations. This mechanism contrasts a more generally established view [50,51] that marine gel phase formation proceeds via cross-linking of negatively charged biopolymers (namely polysaccharides) by Ca^2+^ ions. Only recently, Ding *et al*. [52] reported that phytoplankton EPS can spontaneously self-assemble in calcium-free artificial sea water, forming microscopic gels of 3–4 μm in diameter.

## 3. Experimental Section

### 3.1. Cell Culture

The diatom *Cylindrotheca closterium* (strain CCNA1) was isolated from a seawater sample collected at 12 m depth at the off-shore station SJ108 (12°45′E, 44°45.4′N, 13 November 2006) in the Northern Adriatic. Morphological characteristics and species identification is given in [22]. The diatom was grown in conical flasks containing 100 mL f/2 medium [53]. Cultures were incubated at a temperature of 18 °C, 12:12 dark-light cycle and subcultured every 3–4 weeks. Cells used for AFM experiments were recovered from the stationary growth phase. Cells were counted using a light microscope (Olympus BX51, 200× magnification) with hemacytometer.

**Isolated polysaccharides**. The polysaccharide fraction was isolated from the axenic *C. closterium* culture medium. Operational fractionation and analysis of dissolved polysaccharides were carried out as reported in [16]. Solutions of isolated polysaccharides were freshly prepared prior to AFM imaging. Solution containing 10 mg/L of polysaccharides was prepared in ultrapure water and a 5 μL aliquot of the solution was pipetted directly onto freshly cleaved mica surface and was allowed to dry for 30 mins in an enclosed Petri dish before imaging at ambient conditions.

### 3.2. Macroscopic Gel Phase

**Marine gel material**. Sampling was performed on board of a research vessel during regular cruises along the transect Po River Delta–Rovinj, Istrian coast (Figure 1 in [8]). Scuba divers used 1000 mL plastic cylinders to collect samples of the targeted aggregates. The sampled gelatinous aggregates of over a meter in size, “clouds” [54] (Figure 1b) resided at a 8–20 m depth. The aggregate samples were stored in dark glass bottles and transported to the laboratory within 24 h. The gel phase rinsed with ultrapure water (Milli-Q water) and separated by centrifugation (at 10,000 g, for 10 min at 5 °C) was stored at −20 °C.

The marine gel material used in this study was collected in the summer of 2003 (station SJ 105, 13°10′E, 45°2′N, sampling date June 23, depth 10–13 m) and in the summer of 2004 (station SJ 101, 12°50′E, 45°N, sampling date July 28, depth 20 m). The main difference in the two gel samples collected in two different macroaggregation events (June 2003 and July 2004) was that the macroaggregation process in the Northern Adriatic was more intense and the gelation process was more advanced (older macroaggregates) in July 2004.

**Isolated polysaccharides**. Polysaccharides were extracted from the gel macroaggregates collected in the northern Adriatic Sea in the spring-summer seasons of the mucilage events in the year 2000 (station SJ 310, 13°35′E, 44°35′N, sampling date July 6, depth 15 m) and 2001 (station SJ 105, 13°9′E, 45°2′N, sampling date July 5, depth 14 m). Following the procedure described previously [49] native samples were suspended in 0.2 μm pre-filtered seawater collected at the sampling sites and vigorously stirred for 3–5 h. The suspension was centrifuged at 8000 rpm and the residue resuspended in seawater. Finally, the supernatant solutions were mixed, filtered through 0.45 μm filters and dialyzed against 0.01 M EDTA sodium salt solution and against Milli-Q water (cut-off 12 000 Da) until the complete desalting. The polysaccharide solution was then purified by precipitation upon the addition of isopropanol. The last solution was then dialyzed against Milli-Q water until the conductivity was less than 1 μS/cm before freeze-drying.

The isolated polysaccharide fractions (PS1 and PS2) were studied by light scattering and viscometry. Light scattering measurements were carried out using a Brookhaven Instruments BI200SM photogoniometer with an Innova-70 Argon ion laser as incident source (tuned to 488 nm). Simultaneous linear least squares fitting to both the angular and concentration dependence of scattering data were employed in the Zimm plot analysis. Total counts were recorded over the angular range 30–150°with an EMI-9865A photomultiplier and a Brookhaven BI-2030 correlator. Toluene was used as calibration liquid and the differential refractive index increment was estimated as 0.141 mL/g. Solutions for light scattering measurements were prepared by dissolving the extracted and purified polysaccharides in NaCl solution at a given concentration. Details of solution preparation are given in [49].

Viscometric measurements were carried out by using automatic Schott-Geräte equipment with a Cannon-Ubbelohde suspended level capillary viscometer (diameter 0.53 mm) immersed in a Schott-Geräte water thermostat (25 °C). Solutions were prepared as for light scattering measurements.

### 3.3. AFM Imaging

AFM imaging was performed using a Multimode AFM with Nanoscope IIIa controller (Veeco Instruments, Santa Barbara, CA) with a vertical engagement (JV) 125 μm scanner, using both, contact and tapping modes. Contact mode imaging was performed using silicon-nitride tips (NP-20, Veeco, nom. freq. 56 kHz, nom. spring constant of 0.32 N/m). The force was kept at the lowest possible value in order to minimize the forces of interaction between the tip and the surface. The linear scanning rate was optimized between 1.5 and 2 Hz with scan resolution of 512 samples per line. The tapping mode was applied using silicon tips (RTESP, Veeco, resonance freq. 289–335 kHz, spring constant in the range 20–80 N/m). Ratio of the set point amplitude was maintained to the free amplitude (A/A_0_) at 0.9 (light tapping). The linear scanning rate was optimized between 1.0 and 1.5 Hz with scan resolution of 512 samples per line. Processing and analysis of images was carried out using NanoScope^TM^ software (Digital Instruments, version V614r1). All images presented are raw data except for the first order two-dimensional flattening. Measurements were performed in air, at room temperature and 50–60% relative humidity, which leaves the samples with a small hydration layer, helping to maintain structure [37] using freshly cleaved mica as a substrate.

**Single diatom cells and released polymers**. We used direct drop deposition [37,55] modified for marine phytoplankton samples [22]. A 5 μL volume of the cell culture was pipetted directly onto freshly cleaved mica. Mica slides were placed in enclosed Petri dish for approximately 30–45 mins to allow cells to settle and attach to the surface. Samples were then rinsed three times for 30 s in ultrapure water and placed in enclosed Petri dish to evaporate the excess of water on the mica. Rinsing of samples with ultrapure water was necessary to remove the excess of salts that would hamper AFM imaging under ambient conditions. With this procedure the diatom cells and released polymers stayed firmly attached to the mica surface, enabling stable imaging for AFM experiments.

**Isolated polysaccharides from** ***C. closterium*** **culture**. Solution containing 10 mg/L of polysaccharides was prepared in ultrapure water for AFM imaging. A 5 μL aliquot of the solution was pipetted directly onto freshly cleaved mica surface and was allowed to dry for 30 mins in an enclosed Petri dish before imaging at ambient conditions.

**Marine gel**. Spacemen preparation protocol for AFM imaging was described in detail [21]. The marine gel samples (0.4 cm^3^) were dispersed in ultrapure water in proportion 1:100, under mild stirring for 45 min. 5 μL aliquots of the suspension were pipetted directly onto freshly cleaved mica surface (drop deposition technique) and were allowed to dry for 30 mins in an enclosed Petri dish before imaging.

## 4. Conclusions

Applying high resolution imaging by AFM we demonstrated the capacity of diatom produced polysaccharides to form polymer gel networks by self-assembly. Isolated polysaccharide fractions from marine diatom *C. closterium* culture can form gel network by self-assembly that resembles the gel network of macroscopic gel phase occurring in the northern Adriatic Sea. The process can proceed in pure water in absence of bacterial mediation or interaction with mineral particles. Macromolecular properties of the isolated polysaccharide fraction of marine gel confirmed marked polyelectrolytic behavior allowing a major solubility of polysaccharides in salt solutions favoring chain aggregation and gel formation.

## Figures and Tables

**Table 1.** Comparison of polysaccharide fibril heights.

**Polysaccharide fibrils**	**Number of fibrils analyzed**	**Fibril height/nm**	
		Mean value	Range

Attached to the diatom cell	120	0.85 ± 0.32	0.4–1.8
Marine gel network: early stage	189	0.92 ± 0 .40	0.4–2.0
Marine gel network: mature gel	178	3.58 ± 0.76	1.6–5.0

**Table 2.** Light scattering results at different ionic strength for purified polysaccharides.

**Sample**	**NaCl** **M**	***M*_w_kDa**	***A*_2_×10^6^ cm^3^ mol g^−2^**	***R*_G_ nm**
PS1	0.10	264	−0.41	77
	0.30	145	−0.81	83
	0.50	218	−1.3	99
	0.70	234	−4.8	94
PS2	0.30	222	−0.43	155
	0.70	217	−0.56	131
